# Clinical evaluation of the revolutionizing prosthetics modular prosthetic limb system for upper extremity amputees

**DOI:** 10.1038/s41598-020-79581-8

**Published:** 2021-01-13

**Authors:** Kristin E. Yu, Briana N. Perry, Courtney W. Moran, Robert S. Armiger, Matthew S. Johannes, Abigail Hawkins, Lauren Stentz, Jamie Vandersea, Jack W. Tsao, Paul F. Pasquina

**Affiliations:** 1 Center for Rehabilitation Sciences Research, Henry M. Jackson Foundation for the Advancement of Military Medicine, Bethesda, MD USA; 2 Applied Physics Laboratory, Johns Hopkins University, Laurel, MD USA; 3 Outrider.ai, Golden, CO USA; 4 Uniformed Services University of the Health Sciences, Bethesda, MD USA; 5 Medical Center Orthotics & Prosthetics, Silver Spring, MD USA; 6 University of Tennessee Health Science Center, Memphis, TN USA; 7 Children’s Foundation Research Institute, Le Bonheur Children’s Hospital, Memphis, TN USA

**Keywords:** Biomedical engineering, Musculoskeletal system, Trauma

## Abstract

Individuals with upper extremity (UE) amputation abandon prostheses due to challenges with significant device weight—particularly among myoelectric prostheses—and limited device dexterity, durability, and reliability among both myoelectric and body-powered prostheses. The Modular Prosthetic Limb (MPL) system couples an advanced UE prosthesis with a pattern recognition paradigm for intuitive, non-invasive prosthetic control. Pattern recognition accuracy and functional assessment—Box & Blocks (BB), Jebsen-Taylor Hand Function Test (JHFT), and Assessment of Capacity for Myoelectric Control (ACMC)—scores comprised the main outcomes. 10 participants were included in analyses, including seven individuals with traumatic amputation, two individuals with congenital limb absence, and one with amputation secondary to malignancy. The average (SD) time since limb loss, excluding congenital participants, was 85.9 (59.5) months. Participants controlled an average of eight motion classes compared to three with their conventional prostheses. All participants made continuous improvements in motion classifier accuracy, pathway completion efficiency, and MPL manipulation. BB and JHFT improvements were not statistically significant. ACMC performance improved for all participants, with mean (SD) scores of 162.6 (105.3), 213.4 (196.2), and 383.2 (154.3), *p* = 0.02 between the baseline, midpoint, and exit assessments, respectively. Feedback included lengthening the training period to further improve motion classifier accuracy and MPL control. The MPL has potential to restore functionality to individuals with acquired or congenital UE loss.

## Introduction

The incidence of limb loss has increased in the last decade due to combat injuries in the military population and non-traumatic causes of amputation in the civilian and aging veteran populations such as peripheral vascular disease, diabetes, and malignancy. As of April 2019, 297 service members had sustained upper extremity (UE) limb loss in Operation Iraqi Freedom (OIF) and Operation Enduring Freedom (OEF) alone, representing 17.3% of amputees treated at Military Treatment Facilities (MTFs)^1^. Individuals with UE limb loss experience difficulty performing activities of daily living (ADLs) such as performing acts of personal hygiene (i.e. combing hair, brushing teeth, etc.), dressing, and preparing and eating meals. Prosthetic use following limb loss can restore function and increase quality of life. An ideal UE prosthesis would confer the ability to smoothly manipulate objects in the environment with fidelity, appropriate grip strength, dexterity, anthropomorphism, and fine motor control in a coordinated manner. In this way, the prosthesis would effectively replicate characteristics of the human hand with respect to shape, function, perception, and incorporation into the user’s sense of self^2^. Patients with UE limb loss cited device weight, durability, reliability, dexterity, and limited sensory feedback as factors for UE prosthesis rejection and abandonment^3–5^.

Upper limb prostheses can be divided into passive and active devices. Conventional body-powered prostheses offer simplicity, durability, lightness of weight, and tension feedback to the user with control of basic hand grasps^3^. Active devices—with myoelectric prosthetic hands and neural processing systems—mark a significant technological advancement over conventional body-powered prostheses. Several poly-articulated myoelectric prosthetic hands are currently on the market, including the Michelangelo^®^, i-Limb^®^, and Bebionic^®^ Hands, which enable multiple degrees of freedom (DOF) of control for three to five unique grasps^6^. Commercial hand prostheses are characterized by diminished grip strength and limited finger dexterity and often require manual correction of hand and finger position to switch between grasps, despite being poly-articulated^7^.

In addition to the benefits of poly-articulated upper extremity devices in actuating a greater number of grasps than achievable with body-powered prostheses, progress has been made with respect to prosthetic control schemes. The majority of myoelectric devices rely upon direct control schemes that function according to signal amplitude-based paradigms^8,9^. Myoelectric control classically operates via an on/off paradigm with long training requirements prone to degradation and rarely allows for simultaneous control over multiple DOF. Motion switching is enacted through the control of opposing muscle groups and becomes more difficult with increases in component and motion sequence complexity^10^. Furthermore, these control schemes lack corresponding interfaces through which intuitive motor directives can be connected with movement of the prosthesis in real-time^6^. By contrast, pattern recognition enables users to control multiple DOF simultaneously and independently for more seamless and facile control of multiple movement classes^10^. However, signal degradation may still result due to changes in arm and electrode position, fatigue, and signal cross-talk, with lengthy training requirements^6^. Simon et al. conducted a home trial of trans-radial amputees who controlled multiple-DOF prostheses via a direct control or pattern recognition paradigm and found no significant difference in the number of grasps manipulated between control schemes. However, direct control users spent the majority of their time in a single grasp compared to pattern recognition users, who divided their time between three different grips^9^. The authors theorized that pattern recognition control affords more intuitive selection of and switching between trained motion classes^9^. Hargrove et al. reported similar findings from a randomized controlled trial of trans-humeral amputees with Targeted Muscle Reinnervation (TMR) who performed tasks that required three DOF—the Box and Blocks test, a clothespin relocation task, and a block stacking task—under direct or pattern recognition control schemes^11^. The authors found that real-time pattern recognition control outperformed and was more quickly configured than direct control in these dexterous tasks^11^. Qualitative reports from participants revealed a significant preference for pattern recognition over direct control due to user-perceived ease of use and greater consistency with respect to prosthetic control.

The Modular Prosthetic Limb (MPL) was developed by the Johns Hopkins University Applied Physics Lab (JHU APL) as part of the Defense Advanced Research Projects Agency (DARPA) Revolutionizing Prosthetics initiative. The MPL’s pattern recognition system enables user control over an increased number of actuating joints. The MPL system has 26 independently controllable movement classes (DOF) controlled by 17 independent motors. The system classifies these signals in real-time and allows customization of user movement classes for individual digit, arm and wrist joint control, and coordinated grasping and arm motion. Through this control scheme, a user visualizes and practices moving his or her missing limb, during which the system recognizes and matches the elicited patterns of muscle activation with the selected motion classifier, thus yielding intuitive, personalized, and repeatable prosthetic control^12–14^. The MPL’s control interface utilizes pattern recognition for individual joints and integrated reduced order control (ROC). ROC allows for the combination and integration of complex multi-joint motions to simplify user control over motion classes. An example of ROC with respect to the MPL’s individual finger dexterity allows users to achieve a cylindrical-shaped grasp by visualizing the entirety of the cylindrical grasping motion during training. By training the integrated motion class, users can more easily achieve the target grasp without commanding each finger to close independently to the desired extent (e.g. around a glass) each time. Other unique, innovative features of the MPL include: 360°/s joint speed, multi-articulating fingers, a four DOF thumb, finger spreading via abduction and adduction, joint sensors for position, velocity, and torque, integrated limb control system hardware and software, and a three DOF modular wrist (i.e. rotation, flexion/extension, and abduction/adduction). In addition, the MPL can achieve an anthropomorphic curl strength of 45 ft.-lbs. and maximum elbow joint speeds of 120°/s and includes a three DOF shoulder (i.e. flexion/extension, abduction/adduction, and internal/external rotation) and one DOF elbow^15^. Through these features, the MPL seeks to restore the functionality and kinematics of the human arm and hand to individuals with varied levels of upper extremity limb loss.

Although the MPL can support a modular and diverse set of input control sources, we examined only non-invasive signal acquisition from surface electromyograms (sEMG) as the driver for advanced pattern recognition control. Advances in myoelectric signal processing and TMR have made it possible for individuals with limb loss to identify and discriminate between intended neuromuscular control patterns. TMR is a surgical procedure in which nerves from the residual limb are transferred to other anatomic locations to reinnervate new muscle targets^16^. These residual nerve signals are amplified, allowing for facilitated and more intuitive control of prostheses^16^. The MPL was configured for study use with a socket interface and osseointegration (OI)^17^. OI is the direct joining of living bone and a load-carrying implant, with dynamic interactions at the tissue-implant interface^18^. OI entails the surgical introduction of the implant to bone and bone remodeling through which rigid fixation is achieved and maintained during functional loading^18^.

This study evaluated the performance, feasibility, and acceptance of the MPL in clinical scenarios simulating ADLs. It further sought to assess the number of optimally controlled DOF that can be achieved with this advanced prosthetic control system. There were two study phases: a virtual training phase (Phase I), followed by socket fabrication and electrode integration, and a clinical training phase (Phase II). Through the virtual training sessions, participants were introduced to non-invasive pattern recognition control, which they later utilized to control the physical MPL prosthesis over 12 clinical training sessions. Participants completed formal qualitative and functional assessments during the baseline, midpoint, and final clinical training sessions.

This primary study objective was to evaluate the performance and feasibility of the MPL in clinical scenarios simulating activities of daily living (ADLs). The secondary objective was to determine the number of optimally controlled MPL DOF at different levels of UE loss. We hypothesized that: (1) a greater number of MPL motion classes would be controlled by participants over time, with improvement between the first and last clinical sessions; (2) participants would improve in their ability to perform functional assessments with the MPL between the first and last clinical sessions; and (3) participants will report greater self-perceived ease in performing simulated ADLs with the MPL over their conventional “home” prostheses by the end of the training period due to the MPL’s intuitive control scheme and advanced features.

## Methods

All procedures were performed at Walter Reed National Military Medical Center (WRNMMC) following approval by the WRNMMC Institutional Review Board. All research procedures were performed in accordance with Human Research Protection Program guidelines through the WRNMMC Department of Research Programs (DRP). The study is registered at ClinicalTrials.gov (NCT02887690, 02/09/2016). The study spanned from February 21, 2014 to September 7, 2017. Study length ranged from six to seven weeks depending on the time needed for socket fabrication and integration.

### Participants

This study recruited participants between 18- and 65-years-old with unilateral or bilateral UE loss and was approved to enroll up to 24 individuals in Phase I and 12 individuals in Phase II (Supplementary Materials 1). Recruitment occurred via referral from clinicians at WRNMMC and Medical Center Orthotics & Prosthetics (MCOP).

Participants were included if they could follow study instructions, had no prior history of vertebral disk disease, sciatica, or radiculopathy, and were medically cleared to use a prosthetic device. Participants were excluded if they had traumatic brain injury, a significant Axis I/II diagnosis, a pacemaker, uncontrolled systemic disease, or any other medical condition that might put the subject at risk. Participants were informed of the potential risks and benefits of study participation and their rights as study participants, and given time to ask questions of the study team prior to providing informed consent. Written informed consent was obtained from all participants in accordance with WRNMMC guidelines. Participants were screened using the Test of Memory Malingering in order to ensure adequate participant effort and ability to follow directions; scores lower than 42/50 were exclusionary.

Participants were compensated for their time and travel with a $50 giftcard unless they were an active duty service member or federal employee.

### Phase I: virtual training

Phase I consisted of four, one-hour virtual training sessions. Participants trained with the Virtual Integration Environment (VIE), a virtual reality simulator for UE prosthetic training^19^. An open-source version of the code was created as part of The Open Prosthetics Project (http://openprosthetics.org/) ^20^ in affiliation with the Johns Hopkins University Applied Physics Laboratory (JHU APL) and is available at https://bitbucket.org/rarmiger/minivie. The VIE allows individuals to direct the movements of a virtual avatar using surface electromyogram (sEMG) signals captured from their residual limbs, digitized through an electrically-isolated data acquisition system, and filtered using signal analysis algorithms. Our laboratory has previously described the utility of the VIE in training individuals with upper extremity amputation in this pattern recognition platform for prosthetic control^21,22^. Motions included hand open, wrist flexion, extension, pronation, and supination, several user-selected grasps, “no movement”, and elbow flexion and extension with trans-humeral amputation. Depending on amputation level, electrodes were contained in a non-adhesive Myoband (Thalmic labs, Waterloo, Ontario) or using custom wired bioinstrumentation amplifiers as part of the MPL system. Signal feature extraction and motion classification occurred using machine learning-based pattern recognition software and a Linear Discriminant Analysis classifier. The same software interfaces are used to control the MPL prosthesis^17,21,22^.

At the end of each session, participants completed a 1-DOF Target Achievement Control (TAC)^23^ test that characterized the accuracy with which motion classes were achieved. TAC scores were evaluated with respect to movement completion and path efficiency, which were normalized for the number of grasps trained. Motion completion scores reflect the ability to achieve and maintain a unique pattern of muscle contraction for each motion class over 20 s without signal overlap with other motion class patterns. Path efficiency scores reflect the ability to move a virtual avatar from an initial to target position on the screen. Overshooting and correction for the aberrant motion generates an increased linear motion distance that reduces path efficiency. An average TAC score ≥ 75% was needed to qualify for Phase II.

### Phase II: socket fabrication and clinical training

Participants who qualified for Phase II were cast and fit for a socket that was integrated with sEMG electrodes (Liberating Technology, Inc. Holliston, MA, USA). Electrode number and orientation varied according to amputation level and residual limb anatomy. Participants with OI had previously undergone implantation of the Osseointegrated Prostheses for the Rehabilitation of Amputees implant (Integrum AB, Sweden) and trans-dermal Compress™ system (Zimmer Biomet, Warsaw, Indiana) and received a custom connecting piece to affix the MPL to their implant. These participants used the Myo armband for MPL control.

Participants completed 12 MPL training sessions. Each session included motion class training, ADL simulation, and a TAC assessment. The first (i.e. baseline), sixth (i.e. midpoint), and 12th (i.e. exit) sessions included functional and subjective testing suites. At the baseline assessment, participants completed these suites with their home, conventional prostheses. At the midpoint and exit assessments, these assessments were completed using the MPL. Baseline assessments could not be completed for two participants who lacked home prostheses.

The functional tests administered at the baseline, midpoint, and exit assessments included the Box and Blocks (BB)^24^ test, Jebsen-Taylor Hand Function Test (JHFT)^25^, and Assessment of Capacity for Myoelectric Control (ACMC)^26^. The BB test pertains to efficiency in repeating a motion and grasp sequence to transfer blocks over a raised barrier. The JHFT evaluates the ability to perform seven ADLs. The number of objects moved per second was calculated to track improvements in device control. The ACMC assesses the effectiveness of myoelectric device use during ADLs—dressing, food preparation, ironing, packing a suitcase, or driving—and was developed and validated for UE prosthetic outcomes testing^27^. The suitcase packing ACMC task was used for all participants.

Subjective metrics included the Upper Extremity Functional Scale (UEFS), Trinity Amputation and Prosthesis Experience Scales-Revised (TAPES-R), and modified Visual Analogue Scale/Short-Form McGill Pain Questionnaire (VAS/SF-MPQ) (Supplementary Materials 2)^25,26,28^. The UEFS measures self-reported functional ability to perform 23 ADLs^25^. The TAPES-R captures the type and length of time since amputation, prosthesis usage, and phantom limb pain. The VAS is a simple, internally consistent, and reliable measure of pain validated for clinical and research use^29^. The SF-MPQ is used to assess pain quality and severity^30,31^.

The MPL User Feedback survey (Supplementary Materials 3) was written by our research team for the purposes of this study and assessed participant experiences and perceived levels of activity. Questions were selected based on the recommendations of the UE prosthetic outcome measures working group and prior studies that evaluated user prosthetic experiences, functionality, and self-reported levels of ability with prostheses^17,27,32,33^.

Statistical analysis was performed using repeated measures (RM) one-way ANOVAs and two-tailed t-tests in Prism 8.0 (GraphPad Software Inc., LaJolla, CA). ANOVAs were conducted using Bonferroni adjusted alpha levels of 0.0167 to evaluate the three a priori hypotheses. Remaining analyses were performed with statistical significance defined as p < 0.05.

This effort was supported through Uniformed Services University of the Health Sciences (USUHS) Grant HU0001-11-1-005.

## Results

21 participants were consented. Eight individuals withdrew prior to Phase I due to scheduling concerns. One participant did not meet study criteria to advance to Phase II. One participant withdrew from Phase II due to scheduling conflicts. 11 participants completed all study procedures (Supplementary Materials 1). One participant was omitted due to the use of a translator approved for medical, but not research, services.

Of the 10 individuals included in analyses, 50% had trans-humeral and 50% had trans-radial amputations, including one wrist disarticulation (Table 1). Three individuals with trans-humeral amputations had OI and TMR. Two individuals with trans-radial amputations had congenital limb absence. One individual with congenital limb loss had used a prosthesis daily for ADLs since birth, as reported through the TAPES-R survey, while the other had never used a prosthesis prior to study enrollment.

**Table 1:** Participant demographic and clinical characteristics.

Characteristic	Trans-radial (n = 5)	Trans-humeral (n = 5)	Total
Median age, years (IQR)	29 (21–39)	57 (47–60)	43 (29–57.8)
**MPL configuration**			
Wrist disarticulation	2		
Transradial	3		
Transhumeral		2	
OI/TMR		3	
**Reason for limb loss**			
Trauma	3	4	7
Congenital from birth	2		2
Cancer		1	1
**Affected limb**			
Right	3	1	4
Left	2	4	6
Average time since limb loss (excluding congenital), months (SD)	36.3 (41.4)	123 (40.7)	85.9 (59.5)
Average time of prosthesis use (excluding congenital), months (SD)	5 (6.1)	11.3 (10.3)	8.6 (8.8)

Study participant demographic and clinical characteristics. Of 21 consented research participants, 11 completed all study sessions and 10 were included in final data analysis. Participants with congenital limb absence were excluded from analyses of average time since limb loss and of prosthetic use.

The average time since limb loss and duration of prior prosthetic use, excluding participants with congenital limb loss, were 85.9 months (*s* = 59.5) (Table 1) and 8.6 months (*s* = 8.8), respectively.

### Phase I

12 participants met the TAC cutoff to advance to Phase II, with average scores of 75–100% across motion classes in the Virtual Integrated Environment. After populating the pattern recognition software with sEMG signals corresponding to distinct virtual motion classes, as prompted by on-screen commands, participants performed this one DOF TAC to classify motion accuracy. These participants achieved the requisite scores for study advancement within the allotted four session Phase I study period. Virtual training data was used solely to identify participants with sufficient pattern recognition paradigm mastery to control the physical MPL prosthesis during the clinical training period and were not analyzed as part of this study.

### Phase II

**Figure 1:**
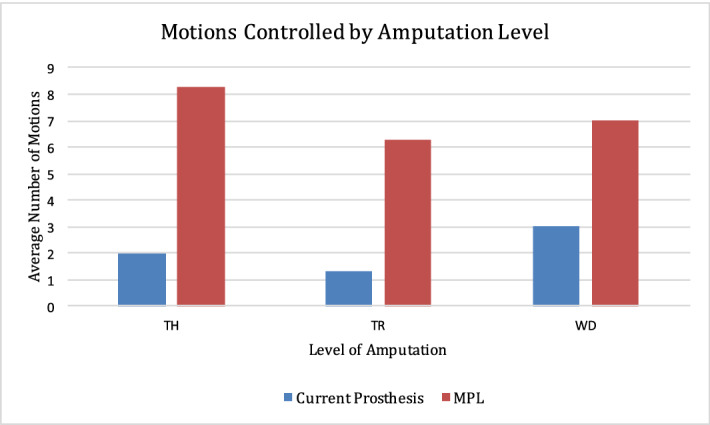
Average number of motions controlled by amputation level, defined as transhumeral, transradial and wrist disarticulation, with participants’ conventional prosthesis (current) compared to the MPL.

Individuals who regularly used a home prosthesis (n = 9) controlled an average of three motion classes at baseline. Home prostheses ranged from conventional devices such as the body-powered hook to myoelectric hands. The initial motion classes trained with the MPL were hand open and “no movement”. On average, participants were able to control six and eight MPL motion classes by the midpoint and exit assessments, respectively. The maximum number of motions controlled with the MPL was 12, including five distinct grasps. There were no significant differences between the number of motions controlled by participants with and without OI/TMR and between amputation levels (Fig. 1).

At the beginning of Phase II, participants were asked about upper extremity motions and activities significant to them that they had not been able to perform satisfactorily with their previous or current prostheses. After participants demonstrated successful manipulation of common objects such as pegs, foam shapes, and stackable cones with the MPL during clinical sessions, they practiced performing ADLs, which were customized according to their expressed task preferences. These ADLs included drinking from mugs, eating with utensils, reaching for overhead objects and stirring pots during meal preparation, writing in cursive, lacing, tying, buttoning a shirt, and playing catch.

TAC scores for individual and combined motion sets improved for all participants across time. A significant improvement in normalized completion scores was observed by RM one-way ANOVA over time, with mean (SD) scores of 3.7 (1.2), 4.1 (1.7), and 6.3 (1.7) at the baseline, midpoint, and exit assessments, respectively, *p* = 0.003. Improvement in normalized path efficiency was also observed by RM one-way ANOVA over time, with mean (SD) scores of 2.5 (0.9), 2.9 (1.1), and 4.2 (1.5), *p* = 0.004, respectively. Simultaneous control of multiple motions slowed task completion by compelling participants to reliably execute distinct patterns of muscle activation and switch between them in an appropriate sequence. Since continuous improvements were observed, the maximum number of controllable motions could not be determined. Participants demonstrated the greatest levels of prosthetic control over the tripod, spherical, tip, and cylindrical grasps.

**Figure 2:**
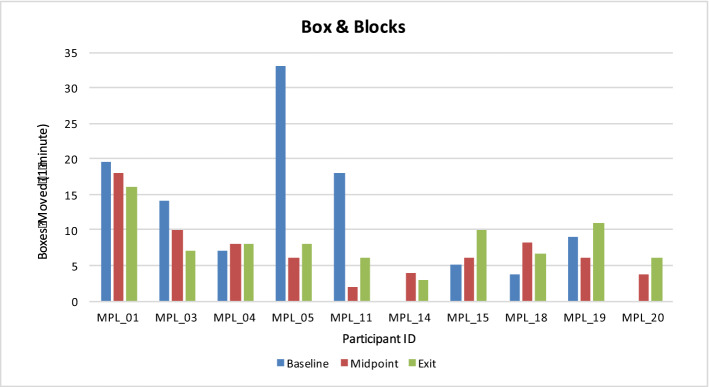
Number of boxes moved during the Box and Blocks assessment for each participant during the baseline, midpoint, and exit sessions. The Box & Blocks task was performed in triplicate during each session and the number of blocks moved was averaged. Two participants (MPL_14 and MPL_20) did not have or regularly use a conventional prosthesis at the time of study participation and did not perform the Box & Blocks task as part of a baseline assessment.

No significant differences were found between the number of BB blocks transferred with the MPL at the exit assessment relative to the number transferred using home prostheses during the baseline session (Fig. 2). BB improvements with the MPL between the midpoint and exit assessments were not significant.

**Figure 3:**
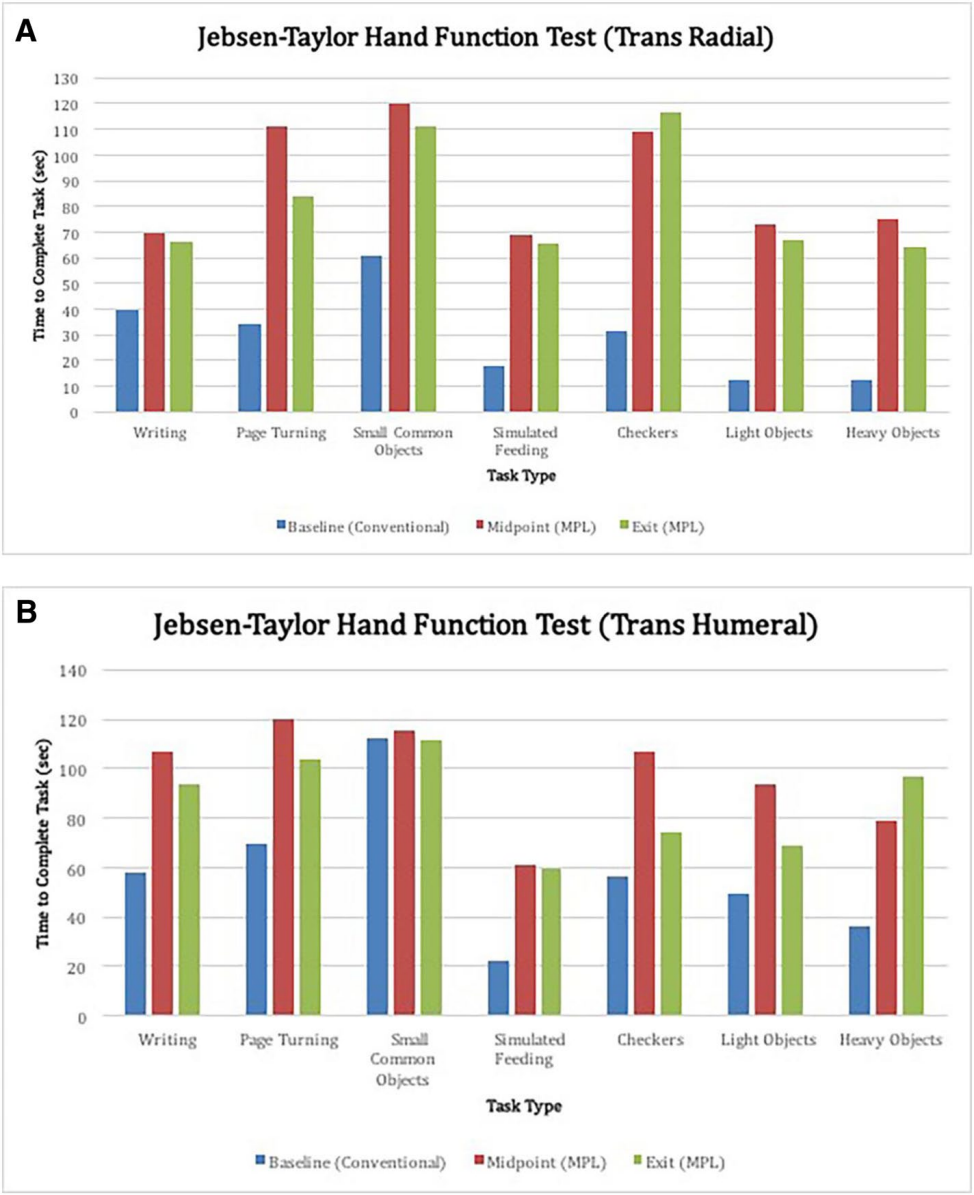
(**A**) The time to complete each task of the Jebsen-Taylor Hand Function Test for participants with trans-radial amputations using the MPL. Participants completed these tasks using grasps and motion classes he or she had trained in previous clinical sessions with the MPL. (**B**) The time to complete each task of the Jebsen-Taylor Hand Function Test for participants with trans-humeral amputations using the MPL. Participants completed these tasks using grasps and motion classes he or she had trained in previous clinical sessions with the MPL.

9 of 10 participants improved in JHFT tasks over time. Individuals with trans-radial amputations outperformed those with trans-humeral amputations with both conventional prostheses and the MPL (Fig. 3a,b). Improvements in JHFT scores over time were not statistically significant between and within individuals (*p* > 0.05). The JHFT tasks with the highest scores were Small Common Objects and Page Turning, while those with the lowest scores were Simulated Feeding and Heavy Objects, regardless of prosthesis used. Of note, the Simulated Feeding task required participants to maintain a steady grip of their choosing on a metal spoon, which was used to scoop individual beans into a can. Participants expressed subjective difficulty in positioning and balancing beans while maintaining their grip on the spoon, which requires sustaining a closed hand grasp motion class throughout the planned motor sequence.

**Figure 4:**
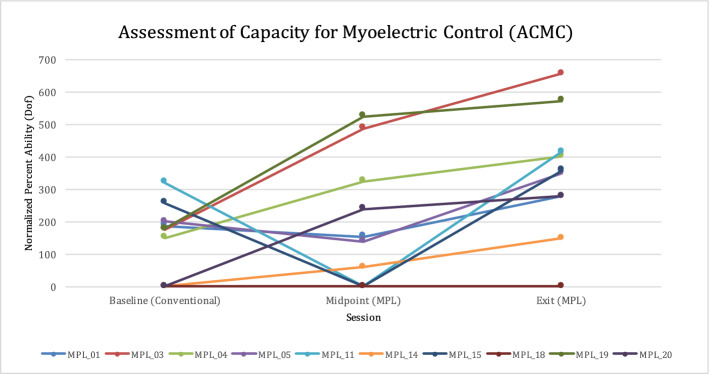
Assessment of Capacity for Myoelectric Control (ACMC) scores for each participant by percent ability, normalized to the number of DOF controlled during the session.

ACMC performance improved for all participants over time by RM one-way ANOVA, with mean (SD) scores of 162.6 (105.3), 213.4 (196.2), and 383.2 (154.3), *p* = 0.02 between the baseline, midpoint, and exit assessments, respectively (Fig. 4). No significant ACMC variation was discerned with respect to age, duration and level of amputation, TAC motion completion or path efficiency scores, or duration of prosthesis use.

UEFS and TAPES-R scores did not vary significantly between the initial and final sessions (*p* > 0.05), including participants with congenital limb absence. Overall, individuals with trans-humeral amputations reported less activity restriction than individuals with trans-radial amputation (mean difference (95% CI) 0.2 (0.007 to 0.4), *p* = 0.01).

Four individuals reported that the MPL was more functional than their current prostheses—which ranged from a body-powered hook to the iLimb^®^ and BeBionic^®^ hands—and cited the ease of changing grasps, ability to pick up larger objects, and durability of the prosthesis as benefits. Three participants felt that at their current level of training (i.e. 12 sessions with the physical prosthesis), the MPL was more useful than their existing prostheses, while three felt that they needed additional training time with the MPL for it to become more useful than their home device. Six participants felt that they needed more practice to reach optimal performance with the MPL in performing routine ADLs and seven participants felt that they could be comfortable using the MPL on a daily basis within two months of study completion. Nine felt that they could complete desired tasks with the MPL if allowed additional time to practice beyond the allotted study period. Most study participants (n = 6) reported that the MPL was heavier than their existing prostheses, decreasing their perceived comfort. The favorite user-identified MPL feature was the ability to complete multiple grasps and articulate individual digits. Additional recommendations for MPL improvement included reduced device weight (n = 4) and access to pause/speed features on the physical device (n = 3).

Six individuals reported that awareness of their phantom limb impacted their performance. Seven of 10 participants imagined moving their phantom limb—without the use of physical visualization aids such as a mirror box—in order to operate the pattern recognition control platform. During sessions in which participants felt as if their phantom was restricted to specific positions (i.e. closed hand), they endorsed greater difficulty in changing grasps and controlling the prosthesis. Neither participant with congenital limb absence reported the presence of a phantom limb or relied upon phantom limb imagery for motion visualization. Participants (n = 4) who reported practicing movement visualization (i.e. different hand grasps and opening the hand) at home without the use of a prosthesis subjectively reported greater ease in controlling the MPL during the next session, though this effect was not reliably and quantifiably demonstrated.

## Discussion

This study is the first clinical evaluation of the MPL in individuals with different levels of UE loss. The MPL seeks to restore the strength, utility, dexterity, and intuitive movement of the human arm and hand to individuals with UE loss. Through modular robotic design, the MPL can be adjusted for any level of amputation and is operated via intuitive, non-invasive, pattern recognition control. All participants improved in MPL control without a plateau in functional and TAC assessment scores. Participants significantly increased their number of executable motions and tasks with the MPL over time. All participants with a home prosthesis manipulated a greater number of motions with the MPL than with their conventional prostheses. Individuals with congenital limb absence were also able to control the MPL using this pattern recognition control paradigm, suggesting that there exist cortical motor mechanisms in the absence of limb development that can be trained for prosthetic control.

Participants expressed significant interest in performing activities with the MPL that they had not been able to achieve since the loss of their limb or birth, in the case of congenital participants. The ADLs participants chose to practice during clinical sessions ranged from practical tasks that may facilitate greater independence such as food preparation and the manipulation of small and common objects, long-held goals—one congenital participant desired to write her name with the hand she had been born without—and a return to activities they had previously enjoyed, such as playing catch or mastering the trigger pose to shoot a gun, in the case of a retired Service member. Two participants expressed fervent interest in re-learning to button shirts and tie shoes. These participants believed that their wardrobe reflected their inability to dress as they desired due to functional limitation, as the acts of buttoning and tying are dependent on coordinated individual digit control not offered by other, prior prostheses. Despite practicing these chosen activities during clinical sessions with the MPL, no significant differences in self-reported functional restriction and the ability to perform standardized ADLs were observed across participants in the qualitative instruments administered. Coupled with the recommendation of the majority of participants that additional time be provided for training with the MPL, it is possible that more time with the prosthesis or more detailed qualitative metrics are needed in order to achieve or capture significant objective and self-perceived functional improvements, respectively.

Although participants with trans-humeral amputation controlled a greater number of motion classes and achieved higher normalized TAC motion completion scores than those with more distal amputations, these differences were not statistically significant. Our observations suggest that the MPL may improve function regardless of amputation level, presence of OI/TMR, and prior prosthetic experience.

MPL_19 had worked with the MPL for 80 h in conference demonstrations prior to this study. This participant underwent trans-humeral amputation secondary to malignancy. The ability to generate mental representations of action may be impaired by residual limb and/or prosthesis disuse^34,35^. Lengthy time gaps in usage may have contributed to regressions in pattern recognition accuracy due to the practice-dependent strength of motor imagery for prosthetic manipulation. No significant differences were observed with respect to MPL_19′s scores and those of the other participants at this level of limb loss.

This study had several limitations, the most notable of which is study size. Study recruitment was hindered by a paucity of both civilian and military patients with UE loss who met study criteria. However, studies have suggested that issues with prosthetic usability can be detected with as few as four to five subjects^36^. In addition, the short duration of the clinical training period was referenced as a limitation upon participants’ ability to achieve his or her full functional potential with the MPL. Participants specifically requested additional time to practice with the VIE and/or MPL at home in order to continue improving in their ability to execute and switch between motion classes and control the physical prosthesis. Two participants likened this suggestion to their practiced visualization of motion classes in Phases I and II during personal downtime at home, such as during commercial breaks or in bed. This served as motivation for the creation of Phase III, through which we will evaluate MPL use in the home setting over 1 year.

The MPL constitutes an advanced UE prosthetic system capable of restoring intuitive dexterous function for individuals with varied levels and mechanisms of UE loss, from blast injuries to congenital limb absence. Participants controlled more motions with the MPL than with their home prostheses after a short training period and continuously improved in pattern recognition accuracy and functional ability.

## Supplementary Information

Supplementary Information 1.

Supplementary Video 1.

Supplementary Video 2.

Supplementary Video 3.

## References

[1] Melcer T (2019). A retrospective comparison of five-year health outcomes following upper limb amputation and serious upper limb injury in the Iraq and Afghanistan conflicts. PM R.

[2] Matrone GC, Cipriani C, Secco EL, Magenes G, Carrozza MC (2010). Principal components analysis based control of a multi-DoF underactuated prosthetic hand. J. Neuroeng. Rehabil..

[3] Biddiss E, Beaton D, Chau T (2007). Consumer design priorities for upper limb prosthetics. Disabil. Rehabil. Assist. Technol..

[4] Biddiss EA, Chau TT (2007). Upper limb prosthesis use and abandonment: A survey of the last 25 years. Prosthet. Orthot. Int..

[5] Carey SL, Lura DJ, Highsmith MJ, Faaop C (2015). Differences in myoelectric and body-powered upper-limb prostheses: Systematic literature review. J. Rehabil. Res. Dev..

[6] Cordella F (2016). Literature review on needs of upper limb prosthesis users. Front. Neurosci..

[7] van der Riet D, Stopforth R, Bright G, Diegel O (2013). 2013 Africon.

[8] Hargrove LJ, Lock BA, Simon AM (2013). Pattern recognition control outperforms conventional myoelectric control in upper limb patients with targeted muscle reinnervation. Annu. Int. Conf. IEEE Eng. Med. Biol. Soc..

[9] Simon AM, Turner KL, Miller LA, Hargrove LJ, Kuiken TA (2019). Pattern recognition and direct control home use of a multi-articulating hand prosthesis. IEEE Int. Conf. Rehabil. Robot.

[10] Kuiken TA, Miller LA, Turner K, Hargrove LJ (2016). A comparison of pattern recognition control and direct control of a multiple degree-of-freedom transradial prosthesis. IEEE J. Transl. Eng. Health Med..

[11] Hargrove LJ, Miller LA, Turner K, Kuiken TA (2017). Myoelectric pattern recognition outperforms direct control for transhumeral amputees with targeted muscle reinnervation: a randomized clinical trial. Sci. Rep..

[12] Bishop W (2008). A real-time virtual integration environment for the design and development of neural prosthetic systems. Conf. Proc. IEEE Eng. Med. Biol. Soc..

[13] Bridges M, Beaty J, Para M, Mashner M, Aggarwal V, Acharya S, Singhal G, Thakor NV (2010). Revolutionizing prosthetics 2009: Dexterous control of an upper-limb neuroprosthesis. Johns Hopkins APL Tech. Digest (Appl. Phys. Lab.).

[14] Hauschild M, Davoodi R, Loeb GE (2007). A virtual reality environment for designing and fitting neural prosthetic limbs. IEEE Trans. Neural Syst. Rehabil. Eng..

[15] Johannes M, Faulring EL, Katyal KD, Para MP, Helder JB, Makhlin A, Moyer T, Wahl D, Solberg J, Clark S, Armiger RS, Lontz T, Geberth K, Moran CW, Wester BA, Van Doren T, Santos-Munne JJ, Peter Walker FJR (2020). Wearable Robotics.

[16] Cheesborough JE, Smith LH, Kuiken TA, Dumanian GA (2015). Targeted muscle reinnervation and advanced prosthetic arms. Semin. Plast. Surg..

[17] Perry BN (2018). Initial clinical evaluation of the modular prosthetic limb. Front. Neurol..

[18] Parithimarkalaignan S, Padmanabhan TV (2013). Osseointegration: An update. J. Indian Prosthodont. Soc..

[19] Armiger R, Tenore FV, Bishop WE, Beaty JD, Bridges MM, Burck JM, Vogelstein JR, Harshbarger SD (2011). A real-time virtual integration environment for neuroprosthetics and rehabilitation. Johns Hopkins APL Tech. Digest.

[20] Prosthetics, O. *Web Tools For Online Collaboration and Study*, <https://openprosthetics.org/projects/jkuniholm/web-tools-online-collaboration-and-study> (2007).

[21] Perry BN (2018). Clinical trial of the virtual integration environment to treat phantom limb pain with upper extremity amputation. Front. Neurol..

[22] Perry BN (2018). Virtual integration environment as an advanced prosthetic limb training platform. Front. Neurol..

[23] Simon AM, Hargrove LJ, Lock BA, Kuiken TA (2011). Target achievement control test: Evaluating real-time myoelectric pattern-recognition control of multifunctional upper-limb prostheses. J. Rehabil. Res. Dev..

[24] Haverkate L, Smit G, Plettenburg DH (2016). Assessment of body-powered upper limb prostheses by able-bodied subjects, using the box and blocks test and the nine-hole peg test. Prosthet. Orthot. Int..

[25] Resnik LM (2012). Reliability and validity of outcome measures for upper limb amputation. J. Prosthet. Orthot..

[26] Lindner HY, Langius-Eklof A, Hermansson LM (2014). Test-retest reliability and rater agreements of assessment of capacity for myoelectric control version 2.0. J. Rehabil. Res. Dev..

[27] Hill W, Hermansson LS, Hubbard S, Stavdahl Ø, Swanson S (2009). Upper limb prosthetic outcome measures (UPLOM): A working group and their findings. J. Prosthet. Orthotics.

[28] Hawker GA, Mian S, Kendzerska T, French M (2011). Measures of adult pain: Visual analog scale for pain (VAS pain), numeric rating scale for pain (NRS pain), McGill pain questionnaire (MPQ), short-form McGill pain questionnaire (SF-MPQ), chronic pain Grade Scale (CPGS), short form-36 bodily pain scale (SF-36 BPS), and measure of intermittent and constant osteoarthritis pain (ICOAP). Arthritis Care Res (Hoboken).

[29] Price DD, Staud R, Robinson ME (2012). How should we use the visual analogue scale (VAS) in rehabilitation outcomes? II: Visual analogue scales as ratio scales: An alternative to the view of Kersten et al.. J. Rehabil. Med..

[30] Melzack R (2005). The McGill pain questionnaire: From description to measurement. Anesthesiology.

[31] Melzack R (1975). The McGill pain Questionnaire: Major properties and scoring methods. Pain.

[32] Organization WH (2002). World Health Organization.

[33] Wright VF (2013). Evidence note: Upper-limb prosthetic outcome measures. Acad. Today.

[34] Chadwell A, Kenney L, Thies S, Galpin A, Head J (2016). The reality of myoelectric prostheses: Understanding what makes these devices difficult for some users to control. Front. Neurorobot..

[35] Malouin F (2009). Effects of practice, visual loss, limb amputation, and disuse on motor imagery vividness. Neurorehabil. Neural Repair.

[36] Virzi R (1992). Refining the test phase of usability evaluation: how many subjects is enough?. Human Fact..

