# Supplementary material for: Clinical evaluation of the revolutionizing prosthetics modular prosthetic limb system for upper extremity amputees
Source: Sci Rep. 2021 Jan 13;11:954. doi: 10.1038/s41598-020-79581-8 (PMC7806748; doi:10.1038/s41598-020-79581-8)
Supplement: Supplementary file 1 — Supplementary Information 1. [file 41598_2020_79581_MOESM1_ESM.docx]

**Supplementary Materials**

Clinical Evaluation of the Revolutionizing Prosthetics Modular Prosthetic Limb System for Upper Extremity Amputees

*Kristin E. Yu^1^, BA; Briana N. Perry^1^, MD; Courtney W. Moran^2^, CPO; Robert S. Armiger^2^, MS; Matthew S. Johannes^3^, PhD; Abigail Hawkins^4^, BA; Lauren Stentz^1^, BA; Jamie Vandersea^5^, CPO; Jack W. Tsao^6,7^, MD, PhD; Paul F. Pasquina, MD, COL (ret)^1,4^

^1^Henry M. Jackson Foundation, Bethesda, MD, USA

^2^Applied Physics Laboratory, Johns Hopkins University, Laurel, MD, USA

^3^Outrider.ai, Golden, CO, USA

^4^Uniformed Services University of the Health Sciences, Bethesda, MD, USA

^5^Medical Center Orthotics & Prosthetics, Silver Spring, MD, USA

^6^University of Tennessee Health Science Center, Memphis, TN, USA

^7^Children’s Foundation Research Institute, Le Bonheur Children’s Hospital, Memphis, TN, USA

Correspondence:

Kristin E. Yu, BA

Center for Rehabilitation Sciences Research

Henry M. Jackson Foundation for the Advancement of Military Medicine

[kyu@hjfresearch.org](mailto:kyu@hjfresearch.org)

**Supplementary Material 1. CONSORT Participant Flow Diagram**

**
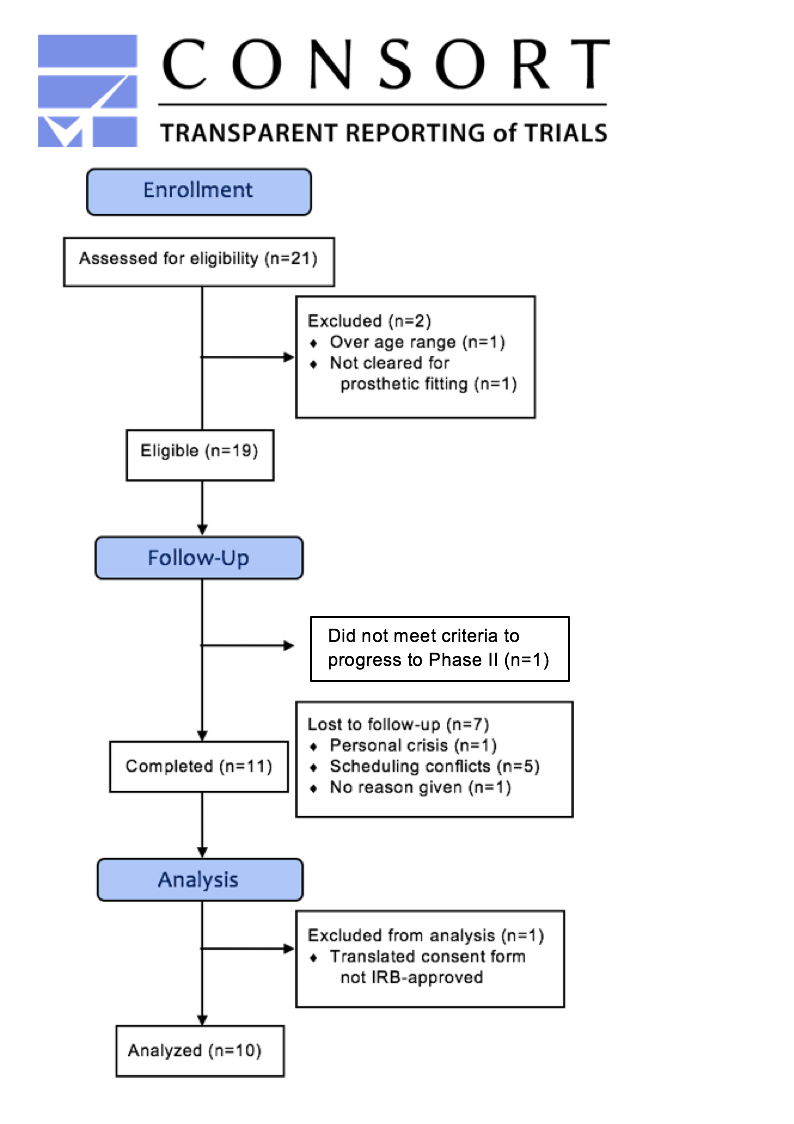
**

**
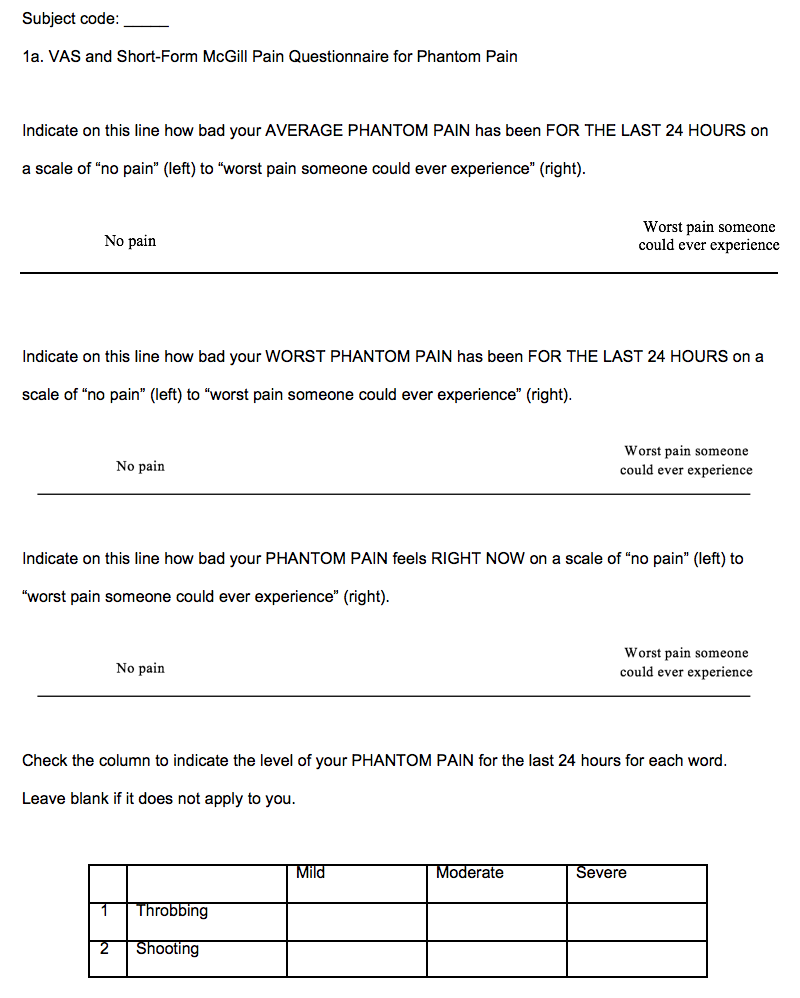
** **Supplementary Material 2: Phantom Limb Pain and Phantom Limb Characteristics**

**
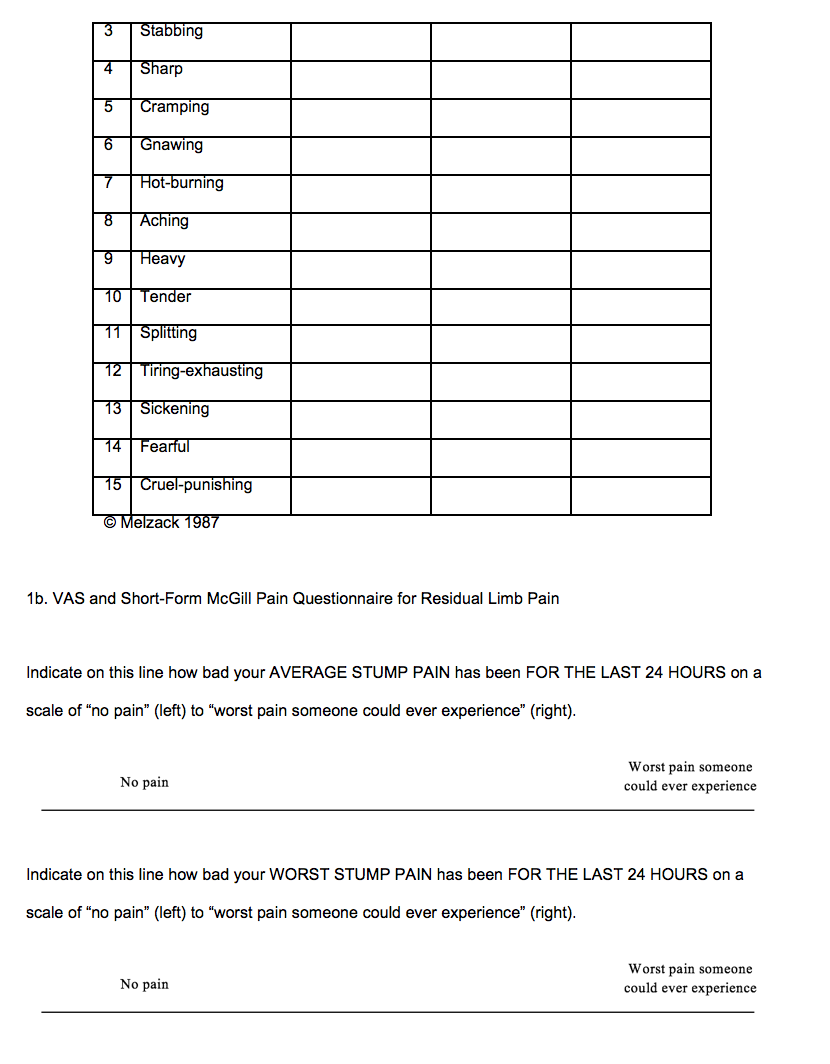
**

**
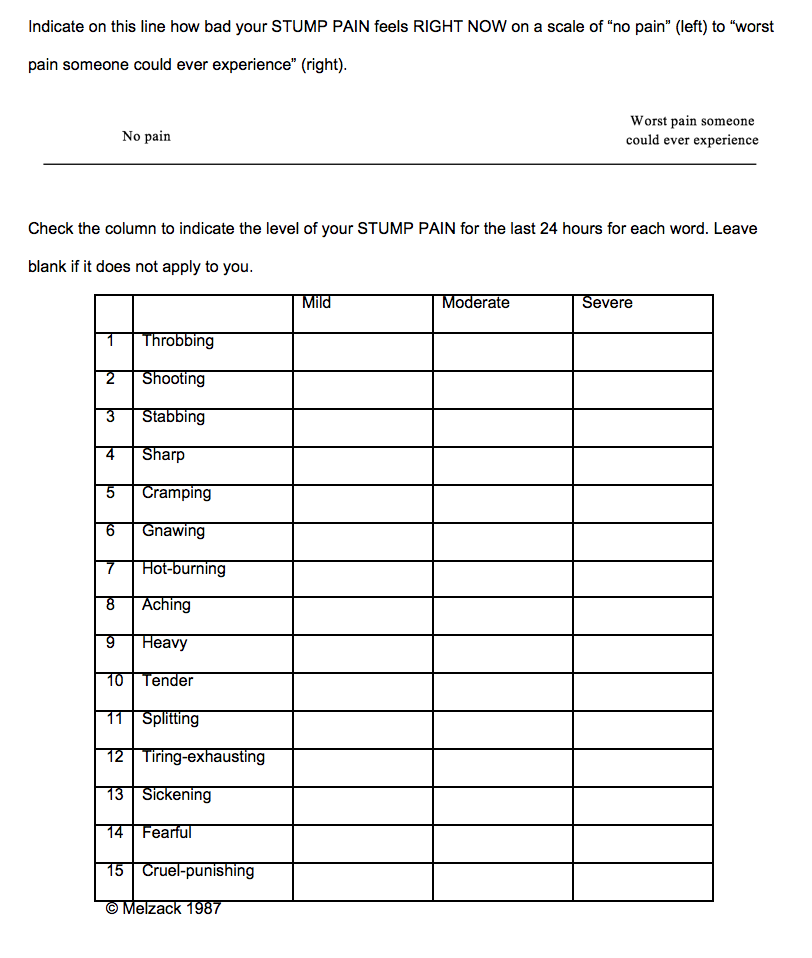
**

**
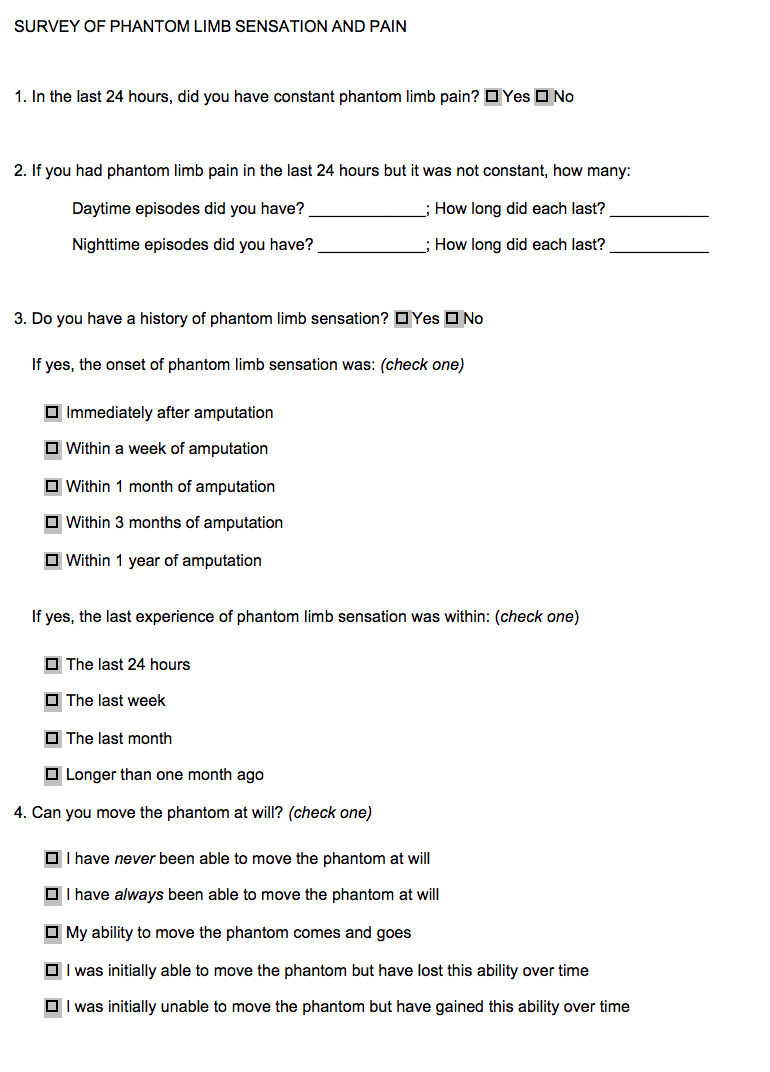
**

**
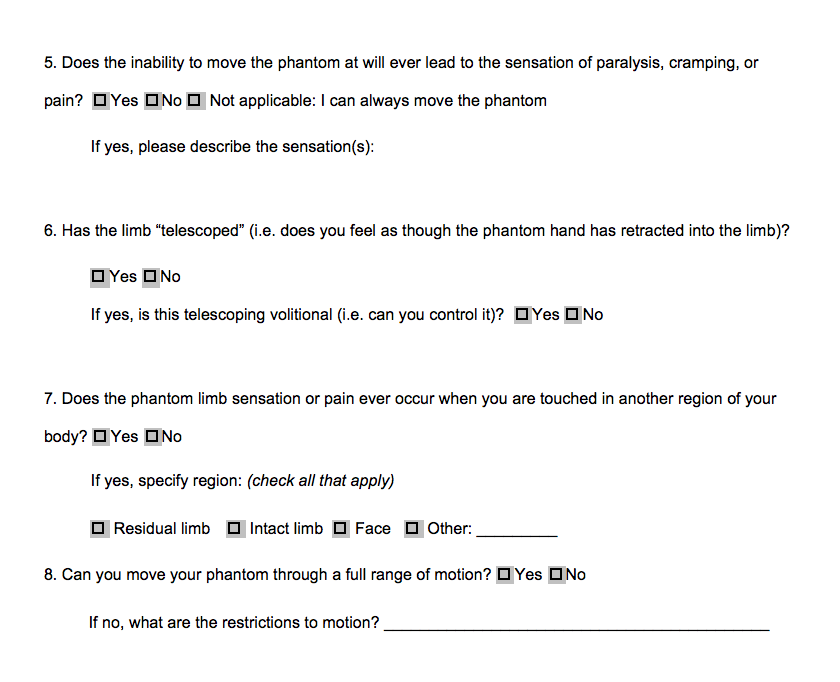
**

**Supplementary Material 3: Modular Prosthetic Limb User Feedback**

**
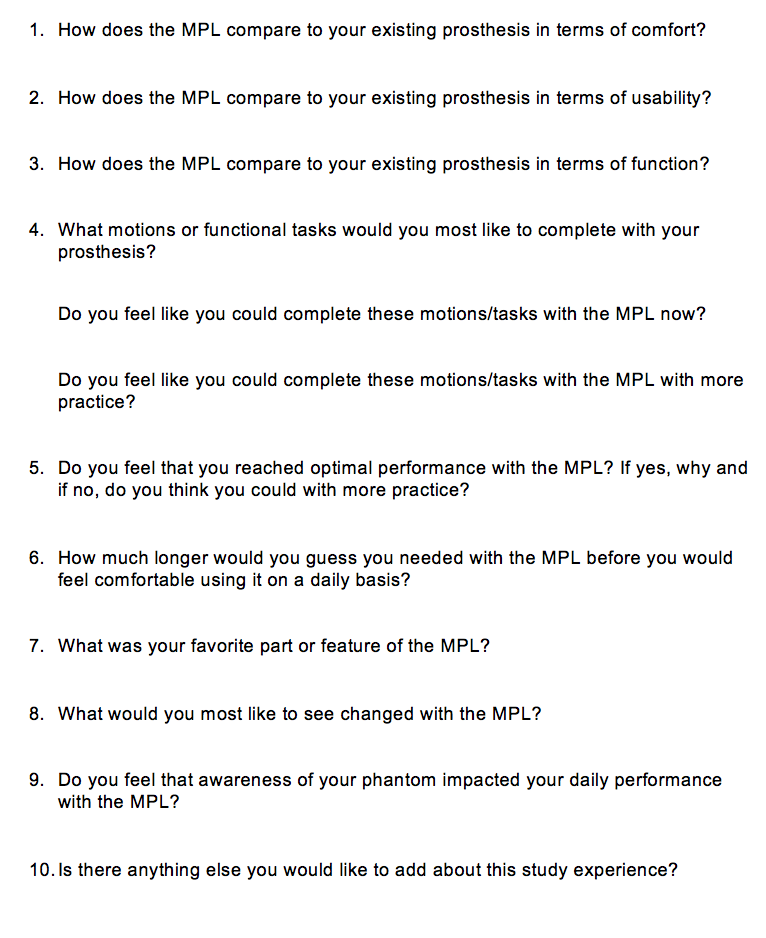
**
